# Clinical characteristics and homology analysis of *Staphylococcus aureus* from would infection at a tertiary hospital in southern Zhejiang, China

**DOI:** 10.1186/s12866-023-02921-x

**Published:** 2023-08-12

**Authors:** Jiarong Zhang, Jingjing Tu, Yongping Chen, Xiaoya Jin

**Affiliations:** 1 Department of Nosocomial Infection Prevention and Control, the First Affiliated Hospital of Wenzhou Medical University, Ouhai District, Wenzhou, Zhejiang 325000 China; 2 The Key Laboratory of Diagnosis and Controlment for the Development of Chronic Liver Disease of Zhejiang Province, Ouhai District, Wenzhou, Zhejiang 325000 China; 3 Department of Infectious Diseases, the First Affiliated Hospital of Wenzhou Medical University, Ouhai District, Wenzhou, Zhejiang 325000 China

**Keywords:** HA-MRSA, CA-MRSA, Drug resistance, Prevalence, Genotyping, Nosocomial infection

## Abstract

**Objective:**

*Staphylococcus aureus* (*S. aureus)*, especially Methicillin resistant *S. aureus* (MRSA), has been disseminated across communities and hospitals, associated with severe infections and organ failure. In order to understand the clinical epidemiological characteristics of *S. aureus* stains in the First Affiliated Hospital of Wenzhou Medical University in 2018, the prevalence and the drug resistance of *S. aureus* stains were investigated, for improving the clinical effective prevention and control of *S. aureus* infection.

**Methods:**

A total of 105 *S. aureus* isolates were separated from wound infection of inpatients in the First Affiliated Hospital of Wenzhou Medical University in 2018, and the department distributions and drug resistance of the isolates were analyzed. The genotyping homology analysis was conducted through the random amplified polymorphic DNA typing (RAPD-PCR) coupled with NTSYS cluster analysis.

**Results:**

Among the 105 strains of *S. aureus*, 31 isolates were MRSA. The prevalence of MRSA among inpatients in the Departments of Burn, Trauma, Orthopedics, Nephrology and Neurosurgery were 35.48%, 19.35%, 9.68%, 6.45%, and 29.03%, respectively. Among the 105 strains, 35.24% strains were the hospital-acquired infections (HAI) and 64.76% strains were community-acquired infections (CAI). DNA genotyping of the 105 *S. aureus* strains showed seventeen different groups, most of which were type I, type VII, type IX, and type VII, the others were scattered.

**Conclusion:**

This study highlights the prevalence of *S. aureus* strains in the First Affiliated Hospital of Wenzhou Medical University in 2018. The emergence and mutation of the strains should be closely monitored for the prevention and control of the *S. aureus* infection and transmission in the nosocomial settings.

## Introduction

*Staphylococcus aureus* (*S. aureus*) is a gram-positive, coagulase-positive and facultative anaerobic organism [1]. *S. aureus* is distributed widely in nature, and it is the main pathogen in host, which mostly colonized in the nasal vestibular mucosa [2], groin [3] and perineum [4] or umbilical cord of the newborn [5]. Through the production of various extracellular secretions including multi-hemolytic toxins, enterotoxins and plasma coagulase, *S. aureus* cause the suppurative inflammations in respiratory tract, urinary tract and surgical site infections [6]. Surgical material implantation [7], prosthesis orthopedics [8] and central venous catheterization [9] are high risk factors for hospital-acquired *S. aureus* bacteremia. Besides, the age, chronic disease, immunodeficiency and genetic susceptibility are also the risk factors [6].

Since the discovery of antibiotics, millions of infected people were saved, and the life expectancies of patients were extended. However, the effectiveness of antibiotics has been challenged by drug-resistant microorganisms. The prevalence of drug resistant microorganism was due to the evolution of dominant drug-resistant strains and the selective role of improper antibiotics usage [10]. In the early 1960s, methicillin-resistant *S. aureus* (MRSA) emerged and spread rapidly in hospital settings worldwide with high morbidity and mortality [11]. The resistance of MRSA was mediated by the *mecA* and *mecC* genes, encoding the enzymes penicillin-binding protein 2a (PBP2a) and PBP2c, respectively. Different from other penicillin binding protein, PBP2a is gifted with low affinity to β-lactams antimicrobial agents, such as penicillinase-labile penicillins (such as penicillin G), penicillinase-stable penicillins (such as methicillin) and cephalosporins (such as cefoxitin)[10].

The severe symptoms of infection and the treatment difficulties caused by MRSA were due to the strong pathogenicity, virulence, and the high drug-resistance [12]. It was reported by the US Center for Disease Control and Prevention (US CDC) that the mortality caused by MRSA was 20,000 approximately in America in 2018, still remained at the highest level in other drug-resistant microorganisms [13]. In Australia, the mortality rate within 30-day was 17.1% in 2018, seriously threatened the public health [14]. In Japan, the length of admission, costs and case fatality rate of MRSA was significantly higher than that of MSSA, concluded in a study from 2016 to 2020 in a tertiary care hospital, indicated the heavy burden of MRSA [15]. In China, although the infection rate of MRSA was decreased from 69% to 2005 to 31% in 2020, it remained at a high level, demonstrated the adverse impact of MRSA on the clinical outcomes and economic burden [16]. In America, the detection rate of MRSA from *S. aureus* in nosocomial settings accounted for 30% ~ 50%. In Asian countries, such as Japan and Singapore, the detection rate of MRSA from *S. aureus* in nosocomial settings has exceeded 50%. The World Health Organization (WHO) has made a list of drug-resistant microorganism posed most threat to the human health in 2017, in which MRSA has been ranked [17, 18].

MRSA was first isolated in the hospital-acquired infection (HAI) patients in the 1960s, and then transmitted in the community-acquired infections (CAI) [19]. MRSA chromosome is integrated with staphylococcal cassette chromosome (SCC) *mec* that is a foreign mobile genetic element including the *mecA* gene conferring resistance against methicillin [20]. Type II and III SCC*mec* elements were carried in typical hospital-acquired MRSA (HA-MRSA) and lack of the Panton-Valentine leucocidin (PVL) toxin gene [21]. The pneumonia, bacteremia, and invasive infections in healthcare-settings were largely related with HA-MRSA infections [22]. Besides, the susceptible population of HA-MRSA were the patients with long-term immunosuppression management or immunocompromised, such as AIDS, aplastic anemia and asplenia patients [23–25], whose immune system were dysfunction or imbalance, as well as those with extended duration of hospital admission [21, 26]. On the contrary, the type IV and V SCC*mec* elements were carried in community-acquired MRSA (CA-MRSA) and carried the PLV toxin gene [27]. The presence of PVL in CA-MRSA caused severe impacts, such as the deep-seated abscess, recurring skin and soft tissue infections episodes, necrotizing pneumonia and sepsis [22, 28]. Furthermore, a great mount of toxins such as phenol-soluble modulins and hemolysins were overexpressed by CA-MRSA, so CA-MRSA exhibits higher virulence than HA-MRSA [29, 30]. The healthy and/or younger population (particular among in children) with fewer related risk factors is susceptible to the infection of CA-MRSA [31]. More recently, the HA-MRSA has been replaced by the CA-MRSA which has been the dominant epidemic strain [32]. Continuous efforts to understand the changing epidemiology of *S. aureus* infection in health-care settings and community are therefore necessary, not only for appropriate antimicrobial treatment and effective infection control but also to monitor the evolution of the species.

Few vaccines or effective chemotherapeutics without side effect to combat MRSA infections was approved, bringing about huge medical and economic burden to the public health [33]. In order to curb the serious consequences of MRSA, it is vital for us to get a comprehensive understanding for clinical epidemiological characteristics of *S. aureus*, especially MRSA, including the prevalence, the drug resistance and genotype of *S. aureus* stains in this hospital. We hope the observations would provide suggestions and strategies for clinical treatment of related infections.

## Materials and methods

### The source of patients

The strains used in this study were isolated from the Departments of Burn, Trauma, Orthopedic, Nephrology and Neurosurgery in the First Affiliated Hospital of Wenzhou Medical University. The study was approved by the ethics committee of the First Affiliated Hospital of Wenzhou Medical University (approval number: no. 2020-089). The demographic characteristics, clinical treatments and so on were collected from an electronic medical record system using a standardized data collection form. Demographic characteristics included age, gender, underlying diseases, length of hospitalization, invasion manipulations. The various system indicators of patients were recorded, including the respiratory system, blood system, liver, cardiovascular system, central nervous system and kidney, etc.

### Bacterial isolates and antimicrobial drug susceptibility tests

A total of 105 *S. aureus* strains were collected from infectious wound sites of patients. The specimens were isolated, cultured and subjected to the drug sensitivity tests in the hospital microbiology laboratory per the local hospital protocol, in accordance with Clinical and Laboratory Standards Institute (CLSI) guidelines (2018) [34]. The isolates were identified by the matrix-assisted laser desorption ionization-time off-light mass spectrometry (MALDI-TOF-MS).

*S. aureus* ATCC29213 was used as quality control. The antimicrobial drug susceptibility patterns of all isolates were determined by the VITEK 2 Compact System, an automatic microbiological analysis system. Isolates were stored at -80 ^o^C in broth contained 30% glycerol for further use.

### Definitions of CA and HA isolates

According to the criteria for identifications of hospital-acquired (HA) and community-acquired (CA) infections issued by the National Health commission of the People’s Republic of China in the year of 2001 (code: [2001]2, refer to the website: http://www.nhc.gov.cn/) and the guidelines raised by the US Centers for Disease Control and Prevention (CDC) as well as the International Nosocomial Infection Control Consortium [35, 36], infections taken in the outpatients or those taken within 48 h of the hospital admission were identified as CA, while the infections taken more than 48 h of the admission were identified as HA [37]. The detailed definitions of HA infection were as following [38]: (i) the infection without a clear incubation period, the signs and symptoms of infection appeared more than 48 h after admission; (ii) the signs or symptoms of a surgical-site infection appeared at admission or started before 48 h after admission; (iii) this infection is directly related to the last hospitalization.

### DNA preparation

For DNA extraction, the isolates cultured overnight were inoculated in 2 mL Luria-Bertani (LB) medium and incubated at 37 ^o^C for 180 rpm until the optical density at 600 nm (OD_600_) reached 0.8 ~ 1.0. The DNA of the bacteria was extracted using Rapid Bacterial Genomic DNA Isolation Kite (Sangon Biotech, Shanghai, China) according to the manufacturer’s instructions. DNA was used immediately or stored at -20 ^o^C for further use.

### The identification of *S. aureus* genotypes

RAPD-PCR was performed according to a previous study with some modifications [39], and the primers (ERIC-1: 5’-ATGTAAGCTCCTGGGGATTCAC-3’; ERIC-2: 5’-AAGTAAGTGACTGGGGTGAGCG-3’) were synthesized by Sangon Biotech for the homology analysis of *S. aureus*. The volume of the reaction system was 50 µL including TaqPCR MasterMix 25 µL (Sangon Biotech (Shanghai) Co., Ltd.), primer 2 µL, bacterial genomic DNA template 3 µL, and ddH_2_O 20 µL. The amplification conditions were as follows: pre-denaturation at 94 ^o^C for 5 min, 94 ^o^C for 1 min, 35 ^o^C for 1 min, 2 min at 72 ^o^C for 40 cycles, and final extension 10 min at 72 ^o^C. The products were verified by 1.5% agarose gel electrophoresis along with a 1500 bp DNA ladder (Sangon Biotech, Shanghai, China) as a DNA size marker.

The produced gels were stained by DNA safe-stain and then photographed by a gel documentation system (Bio-Rad, USA). To compare the isolates, cluster analysis was performed using the Dice algorithm and unweighted pair group method with arithmetic mean (UPGMA) type. According to the requirements of computer analysis, the reproducible stripes from each addition are graded as unit characters. For the pairing comparison and the estimation of the genetic relationship, the binary data obtained by the marker system were analyzed separately by Dice’s coefficient. The genetic similarity coefficient was calculated by Dice coefficient (*S*_*D*_) with NTSYS software. The UPGMA was carried out for cluster analysis by NTSYS software. If the *S*_*D*_ was ≥ 80%, the clones were recognized as the same RAPD type. Based on number and weight from the electrophoresis bands, the genotypes were sort out [40].

### SOFA scores

The Sequential Organ Failure Assessment (SOFA) score was used to describe the dysfunction or failure of single organ with a sequential way, and evaluate the degree of organs from mild dysfunction to severe failure [41]. Six organs system prone to dysfunction clinically were included, which were respiratory, circulatory, kidney, liver, nerve and cardiovascular system. Table 1 showed the scoring rules according to the literature [42]. A higher SOFA score was associated with an increased probability of mortality [43].

**Table 1:** The detailed scoring rules

Variable	Scored 1	Scored 2	Scored 3	Scored 4
**Respiratory system** (PaO_2_ / FiO_2_ mmHg)	˂ 400	˂ 300	˂ 200	˂ 100
**Circulatory system** (platelet * 10^3^/mm^3^)	˂ 150	˂ 100	˂ 500	˂ 20
**Kidney** (creatinine µM/urine volume)	110–170	171–299	300–440 or ˂ 500 mL / d	> 440 or ˂ 200 mL / d
**Liver** (serum bilirubin µM)	20–32	33–101	102–204	> 204
**Central nervous system** (Glasgow coma score)	13–14	10–12	6–9	˂ 6
**Cardiovascular system** (blood pressure)	Mean arterial pressure ˂ 70 mmHg	Dopamine ≤ 5 or dobutamine (any dose)	Dopamine > 5 or adrenaline or noradrenaline ≤ 0.1	Dopamine > 15 or adrenaline or noradrenaline > 0.1

### Statistical analysis

In all analyses, *P*-values ≤ 0.05 were considered significant. To analyze categorical variables, a χ^2^ or two-tailed Fisher’s exact test was used as appropriate. Continuous variables were analyzed by using the Student *t*-test for normally distributed variables or the Mann-Whitney U-test for variables that are not normally distributed.

## Results

### The compositions, sources and distributions of strains

**Fig. 1:**
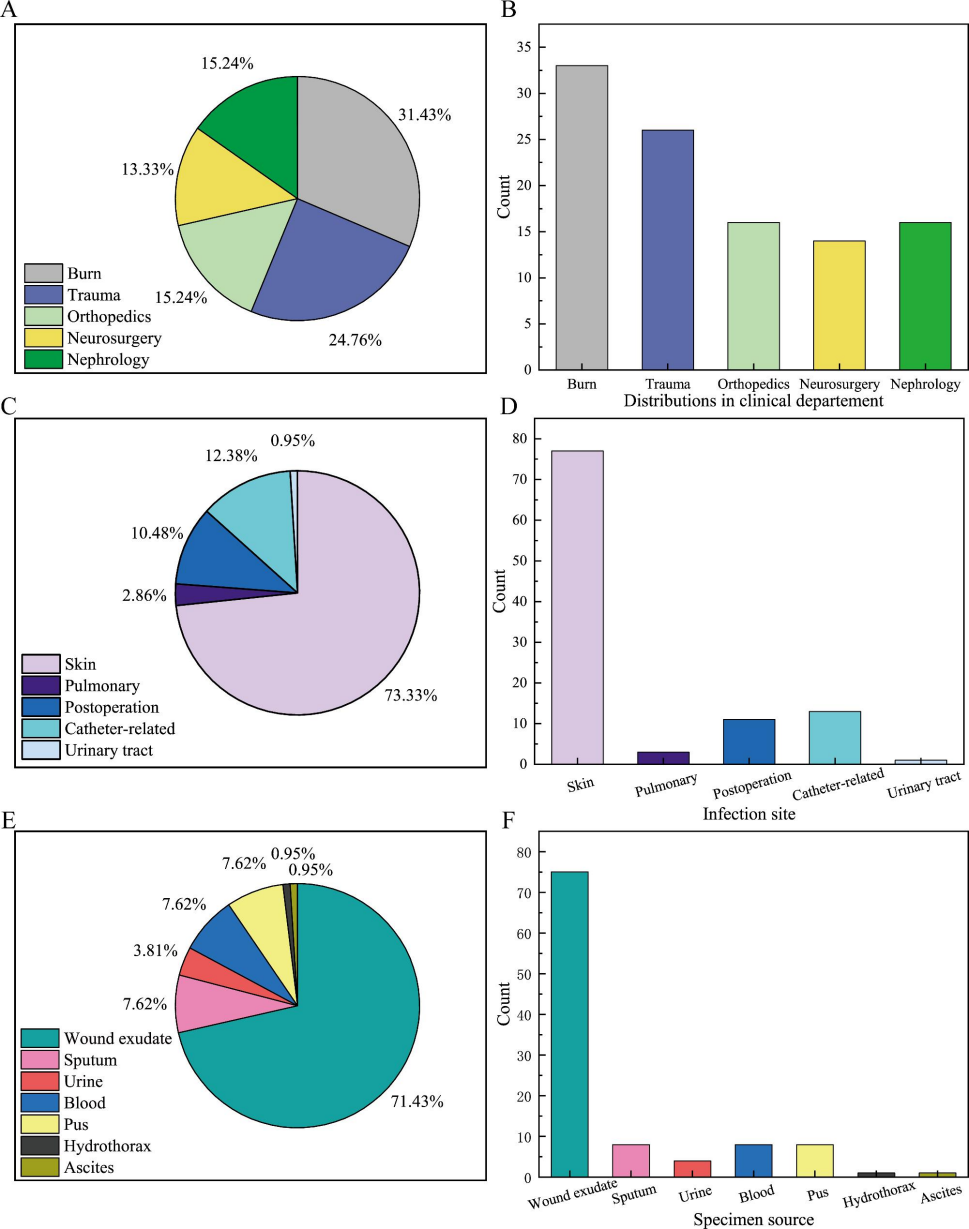
The distributions of *S. aureus* strains in clinical departments (**A ~ B**), infection sites (**C ~ D**) and specimen sources (**E ~ F**)

There were 105 *S. aureus* strains collected from inpatients in 5 clinical departments, and the detailed information were displayed in Table 2. Wherein, 33 strains were from the Burn Department, 26 strains from the Trauma Department, 16 strains from the Orthopedics Department, 14 strains from the Nephrology Department and 16 strains from the Neurosurgery Department (Fig. 1A **~ B**). According to the infective sites, the strains were divided into 5 groups, which were skin, pulmonary, postoperation, catheter-related and urinary tract infections (Fig. 1C **~ D**). Among these groups, the proportion of skin infections were highest, up to 73.33%. However, the proportion of urinary tract infections were lowest. According to the specimen source, the strains could be separated into 7 groups (Fig. 1E **~ F**). The most of strains were from the wound exudate, and the minority of strains were from the hydrothorax and ascites.

**Fig. 2:**
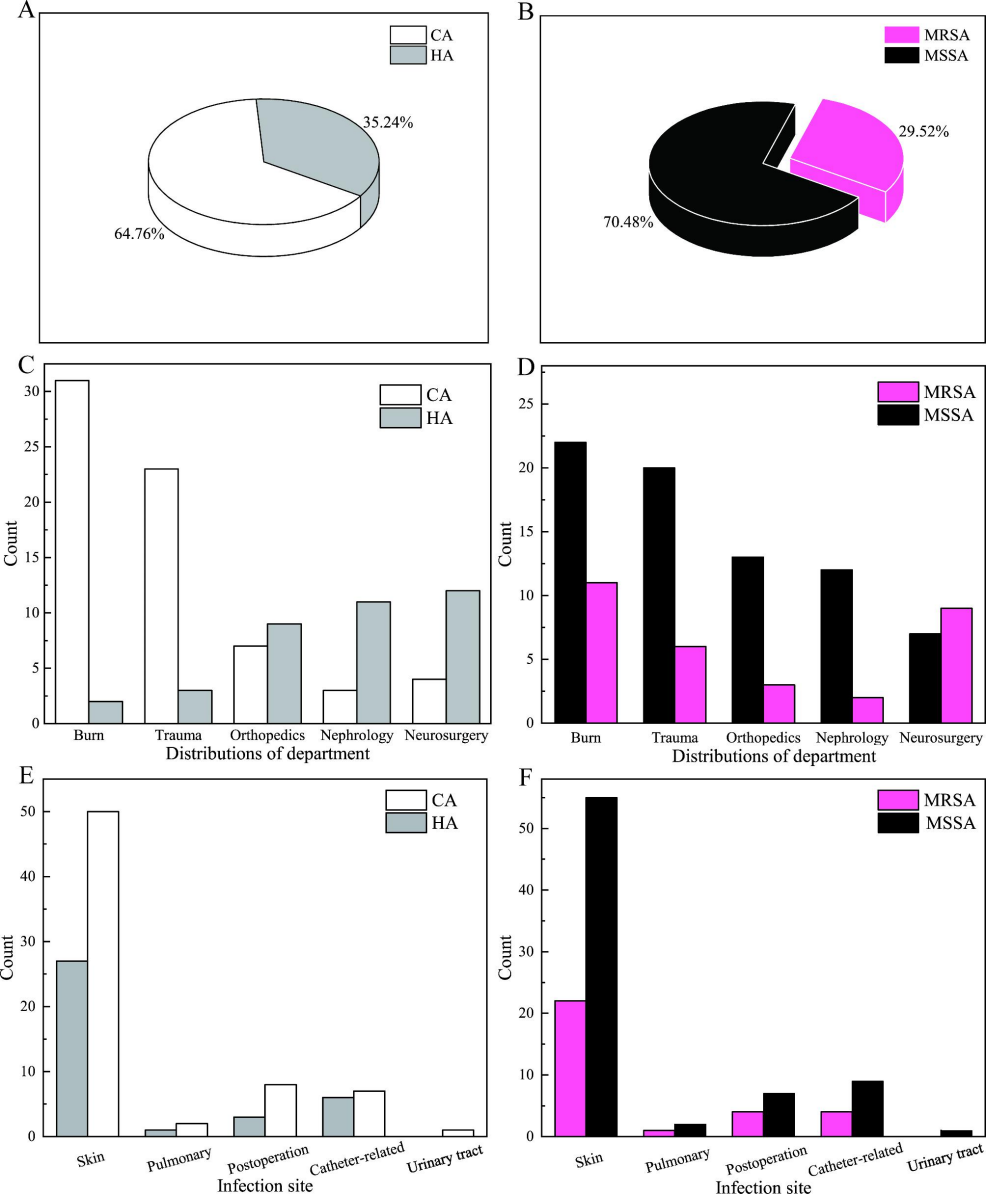
The composition, distributions in clinical department and infection sites between CA and HA infections (**A, C, E**) and between MRSA and MSSA infections (**B, D, F**)

Among 105 episodes, 68 strains were classified as CA groups (64.76%) and 37 strains were classified as HA group (35.24%), shown in Fig. 2A. Most of CA infections were in the Burns and Trauma Department, while most of HA infections were in Orthopedics, Nephrology and Neurosurgery, shown in Fig. 2C. Additionally, the distributions of CA groups in the five infection sites were higher than that of HA groups (Fig. 2E).

There were 31 strains MRSA detected (29.52%), of which 30 strains were resistant to cefoxitin and oxacillin, and 1 strain was resistant to cefoxitin but sensitive to oxacillin, as shown in Fig. 2B. Among 31 strains of MRSA, 35.48% were isolated from the Burn Department, 19.35% in the Trauma Department, 9.68% in Orthopedics, 6.45% in Nephrology, and 29.03% in Neurosurgery, shown in Fig. 2D. Few differences were observed in the department distributions between MSSA and MRSA group, as shown in Table 3. In the 5 infection sites, most of MRSA strains were isolated from skin infections, and second were from catheter-related as well as postoperative infections, but few isolated from urinary tract (Fig. 2F).

**Table 2:** The information of inpatients with *S. aureus* infection

patient characterization	total (n = 105)	MSSA (n = 74)	MRSA (n = 31)	HA (n = 37)	CA (n = 68)
age (median)	59	61	53	57	62
< 45	23	17	6	8	15
45 ≤ age < 60	30	18	12	13	17
60 ≤ age < 75	40	29	11	11	29
> 75 years old	12	10	2	5	7
**gender**	\	\	\	\	\
male	69	49	20	27	42
female	36	25	11	10	26
**the amount of underlying illness (median)**	1	2	0	0	1
0	49	31	18	19	30
1	30	24	6	12	18
2	21	15	6	5	16
≥ 3	5	4	1	1	4
**length hospitalization (median days)**	**18**	**22**	**15**	**10**	**21**
< 3 days	2	2	0	2	0
3 ≤ days < 7	9	9	0	6	3
7 ≤ days < 14	28	23	5	17	11
14 ≤ days < 30	49	31	18	12	37
> 30 days	17	9	8	0	17
**received invasion manipulations (total)**	**25**	**16**	**9**	**8**	**17**
surgical procedure	11	7	4	3	8
arteriovenous catheterization	8	5	3	3	5
indwelling catheter	1	0	1	0	1
tracheal cannula	2	1	1	1	1
hemodialysis	2	2	0	1	1
puncture	1	1	0	0	1
**the amount of received antibiotic types (total)**	**105**	**74**	**31**	**37**	**68**
0	7	5	2	1	6
1	50	39	11	20	30
2	38	23	15	11	27
≥ 3	10	7	3	5	5

**Table 3:** Comparison of MRSA and MSSA in different departments

	Burn	Trauma	Orthopedics	Neurosurgery	Nephrology	Total	*P*
MSSA	22	20	13	7	12	74	0.09
MRSA	11	6	3	9	2	31	
Total	33	26	16	16	14	105	

### Drug resistance of strains

**Fig. 3:**
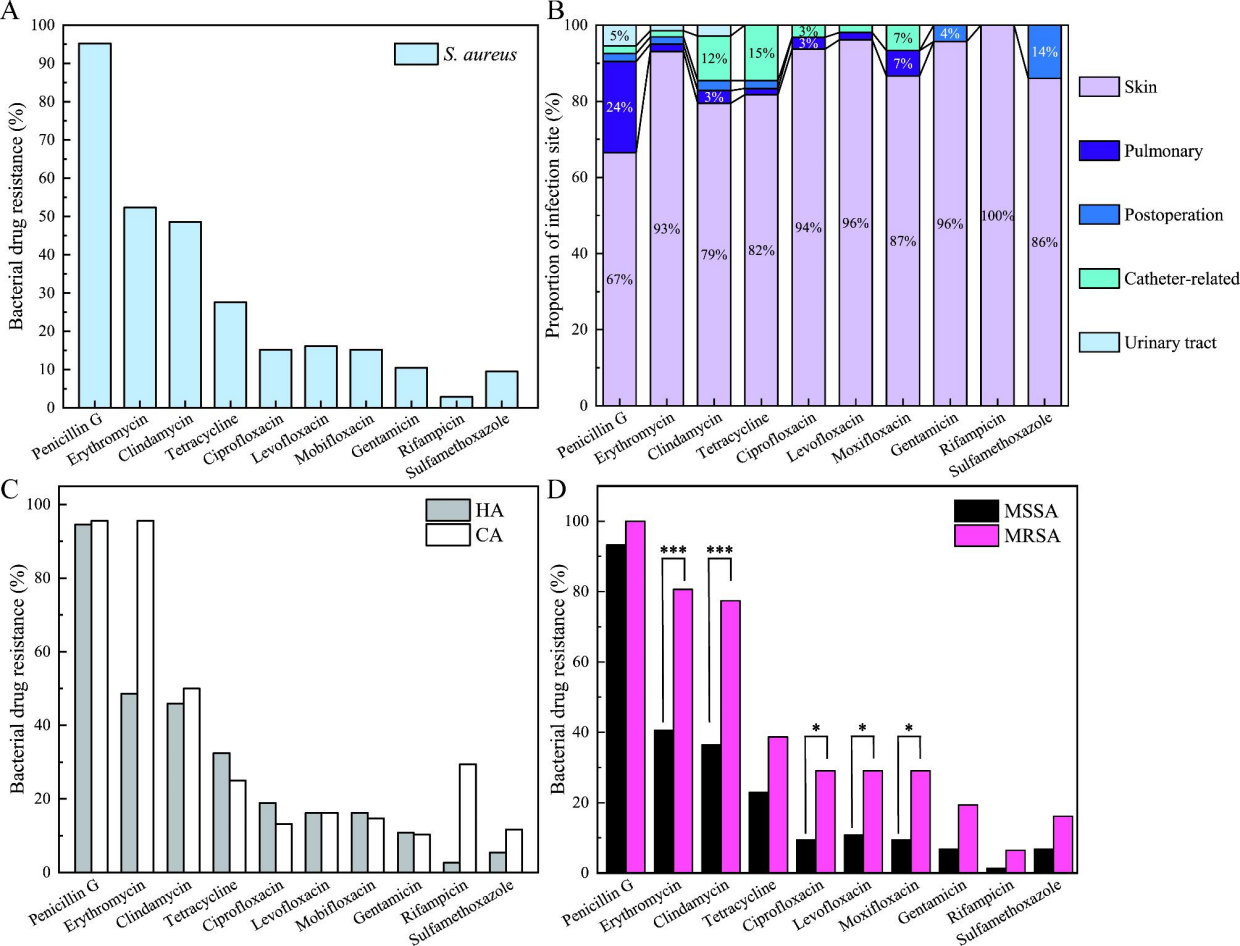
Bacterial drug resistance in *S. aureus* and the distributions in different infection sites (**A ~ B**). The comparisons of bacterial drug resistance between HA and CA group and between MRSA and MSSA group (**C ~ D**).

In vitro antimicrobial susceptibilities, *S. aureus* were resistant to Penicillin G (95.23%), Erythromycin (52.38%), Clindamycin (48.57%), Tetracycline (27.62%), Ciprofloxacin (15.24%), Levofloxacin (16.16%), Moxifloxacin (15.23%). The resistant rate to Gentamicin, Sulfamethoxazole, and Rifampicin were 10.48%, 9.52%, and 2.86%, respectively. The resistance of isolated strains to Nitrofurantoin, Tegacycline, Vancomycin, Teicoplanin, Linezolid and Quinu/Dafoptin was not found (Fig. 3A). The drug resistance of the strains in different infection sites were diverse (Fig. 3B). In the skin infections, the bacterial resistance of strains to Rifampicin was found the highest one (100%), and Levofloxacin along with Gentamicin was the second highest one (96%). In the pulmonary infections, the bacterial resistance to Penicillin G was found the highest (24%), and then was Moxifloxacin, accounted for 7%. In the postoperative infections, the bacterial resistance to Sulfamethoxazole was the highest (14%), but little resistance to Ciprofloxacin, Levofloxacin and Moxifloxacin was found. In the catheter-related infections, the resistance of strains to Tetracycline was the highest, accounted for 15%. In the urinary tract, the resistance of strains to Penicillin G was the highest, accounted for 5%.

The results of bacterial drug resistance in CA and HA infections were shown in Fig. 3C. In HA infections, the bacterial resistance of strains to Penicillin G was the highest one, and the second was Erythromycin, but the lowest one was Rifampicin. In CA infections, the bacterial resistance of strains to Penicillin G and Erythromycin was the highest (95.60%), and lowest one was Gentamicin (10.32%). There was no significant difference in drug resistance between the HA group and the CA group (Fig. 3C; Table 4).

The results of bacterial drug resistance in MRSA and MSSA infections were shown in Fig. 3D. In MRSA infections, the bacterial drug resistance to Penicillin G was the highest (100%), and second was Erythromycin (80.65%), but the drug resistance to Rifampicin was the lowest (6.45%). In MSSA infections, the resistance of strains to Penicillin G was highest (94.24%), and the drug resistance to Rifampicin was the lowest (1.35%). For comparison, it found that the resistance rate of MRSA to Erythromycin, Clindamycin, Ciprofloxacin, Levofloxacin and Moxifloxacin tested in this survey was significantly higher than that of MSSA strains (Table 5).

**Table 4:** Comparison of drug resistance of *S. aureus* between HA group and CA group

Drug	Drug resistance of *S. aureus*		χ^2^	*P*
	HA Group(n = 37)	CA Group(n = 68)		
Penicillin G	94.60% (35/37)	95.60% (65/68)	0.00	0.98
Erythromycin	48.65% (18/37)	54.41% (37/68)	0.32	0.57
Clindamycin	45.95% (17/37)	50.00% (34/68)	0.16	0.69
Tetracycline	32.43% (12/37)	25.00% (17/68)	0.66	0.42
Ciprofloxacin	18.92% (7/37)	13.23% (9/68)	0.60	0.44
Levofloxacin	16.2% (6/37)	16.2% (11/68)	0.00	1.00
Moxifloxacin	16.22% (6/37)	14.71% (10/68)	0.04	0.84
Gentamicin	10.81% (4/37)	10.29% (7/68)	0.01	0.93
Rifampicin	2.70% (1/37)	2.94% (2/68)	0.00	0.94
Sulfamethoxazole	5.40% (2/37)	11.76% (8/68)	0.51	0.48

**Table 5:** Comparison of drug resistance of *S. aureus* between MSSA and MRSA

Drug	MSSA (n = 74)	MRSA (n = 31)	χ^2^	*P*
Penicillin G	69 (93.24%)	31 (100.0%)	/	0.32
Erythromycin	30 (40.54%)	25 (80.65%)	14.09	0.00
Clindamycin	27 (36.49%)	24 (77.42%)	14.65	0.00
Tetracycline	17 (22.97%)	12 (38.71%)	3.09	0.08
Ciprofloxacin	7 (9.46%)	9 (29.03%)	6.48	0.01
Levofloxacin	8 (10.81%)	9 (29.03%)	5.25	0.02
Moxifloxacin	7 (9.46%)	9 (29.03%)	6.48	0.01
Gentamicin	5 (6.76%)	6 (19.35%)	/	0.08
Rifampicin	1 (1.35%)	2 (6.45%)	/	0.21
Sulfamethoxazole	5 (6.76%)	5 (16.13%)	/	0.12

### Length of hospitalization

**Fig. 4:**
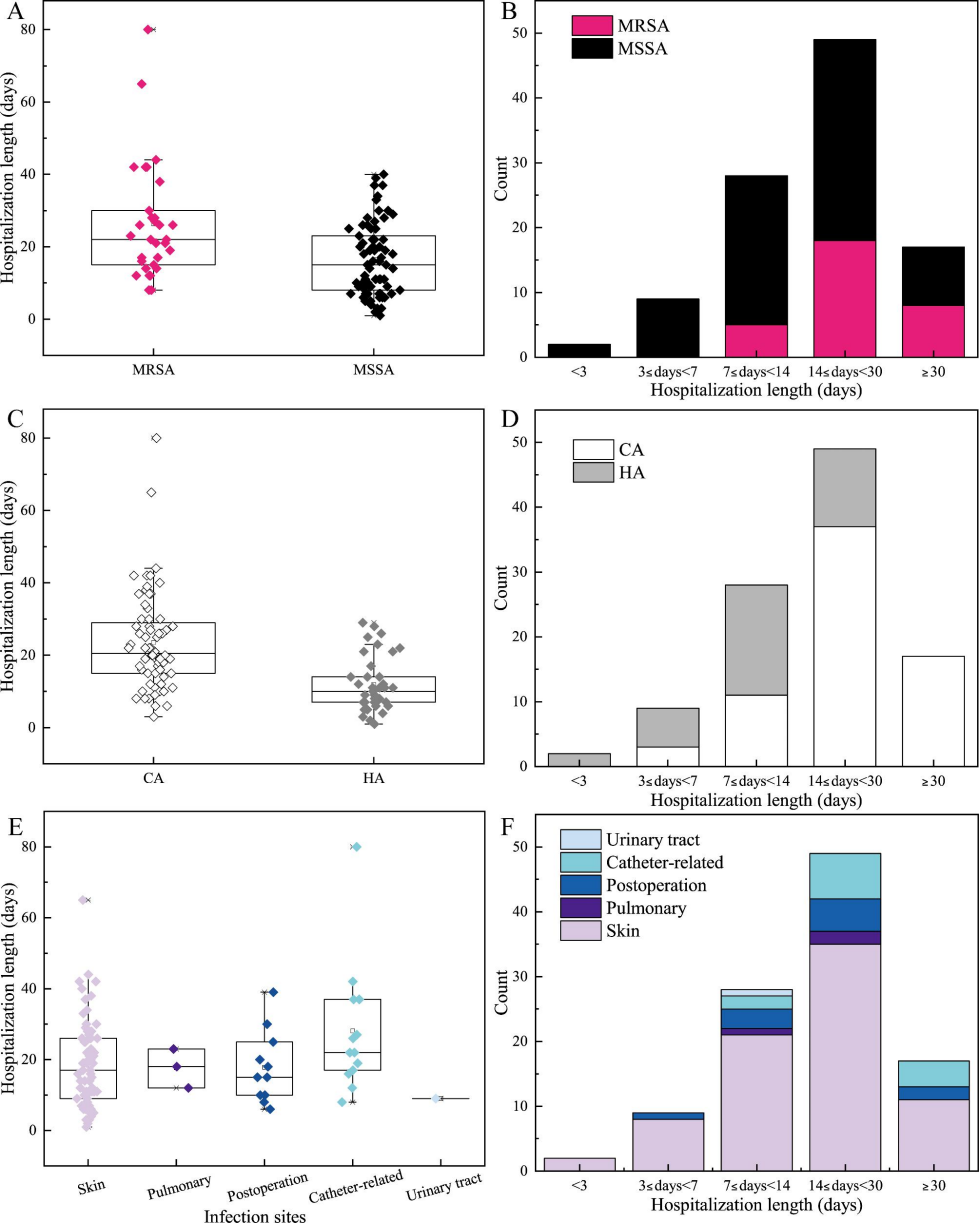
Hospitalization length in the groups of MRSA and MSSA (**A ~ B**), CA and HA (**C ~ D**), and different infection sites (**E ~ F**).

The median of hospitalization length in MRSA infections (22 days) were longer than that of MSSA (15 days) (Fig. 4A), and the hospital length for most MRSA and MSSA infections was 14 to 30 days (Fig. 4B). The median of hospitalization length in CA infections were longer than that of HA (Fig. 4C), which were 21 days and 10 days respectively. The duration of hospitalization in most CA inpatients was 14 to 30 days, but in most HA infections was 7 to 14 days (Fig. 4D). The hospitalization length longer than 30 days were all CA infections, and the length shorter than 3 days were all HA infections. The median of hospitalization length in 5 infection sites were diverse (Fig. 4E). The longest one was catheter-related infections (28 days), and the lowest was urinary tract infections (9 days). The median of hospitalization length in skin, pulmonary and postoperative infections were 17 days. The duration of hospitalization shorter than 3 days was occurred in skin infections, and longer than 30 days was occurred in skin, postoperative and catheter-related infections (Fig. 4F).

### SOFA score

**Fig. 5:**
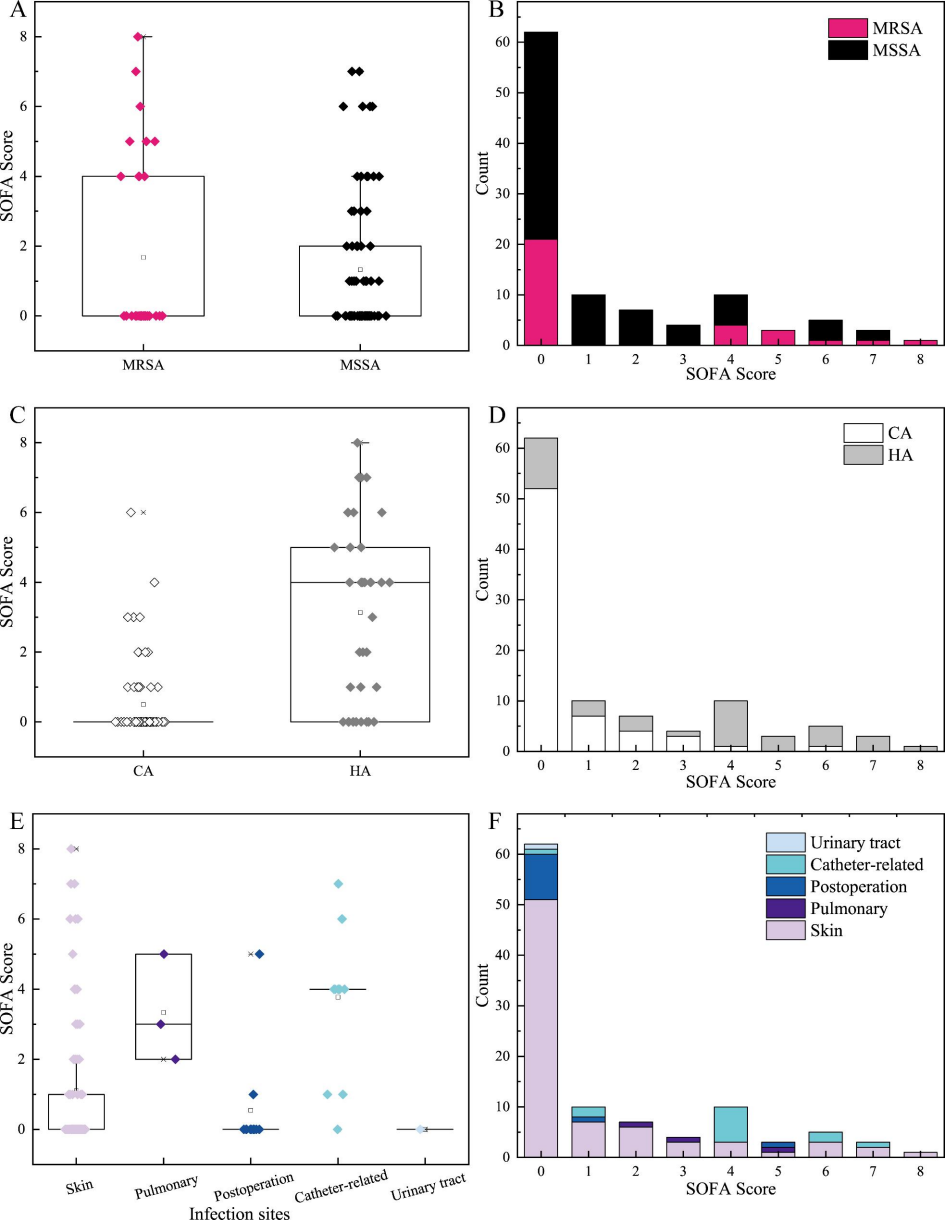
The median and distributions of SOFA score between MRSA and MSSA infections (**A ~ B**), between HA and CA infections (**C ~ D**) and among 5 infection sites (**E ~ F**).

The median of SOFA score in MRSA was higher than that of MSSA, which scored 2 and 1 respectively (Fig. 5A). It found that the most infections of MRSA and MSSA were scored 0, and the highest SOFA score of 8 was MRSA infections (Fig. 5B). The median SOFA score in CA infections were lower than that of HA infections (Fig. 5C). From Fig. 5D, it found that the infections who scored high SOFA (≥ 4) were most of HA group, while the infections who scored lower SOFA (< 4) were most of CA group. The difference of median SOFA score in 5 infection sites was observed, in which the SOFA of catheter-related infections scored the highest, and the urinary tract scored the lowest (Fig. 5E). From Fig. 5F, it found that the skin infections distributed in all of 8 SOFA levels, and the highest score of 8 only observed in skin infections. Besides, the SOFA score of pulmonary infections mainly distributed in the levels of 1, 2, 3, 5, in which the most infections scored 5. Next in the postoperative and urinary tract infections, the SOFA scores of patients were 0. Finally, in the catheter-related infections, the most patients were scored 4, and others were scattered in the score of 1, 6 and 7.

### RAPD homology analysis

**Fig. 6:**
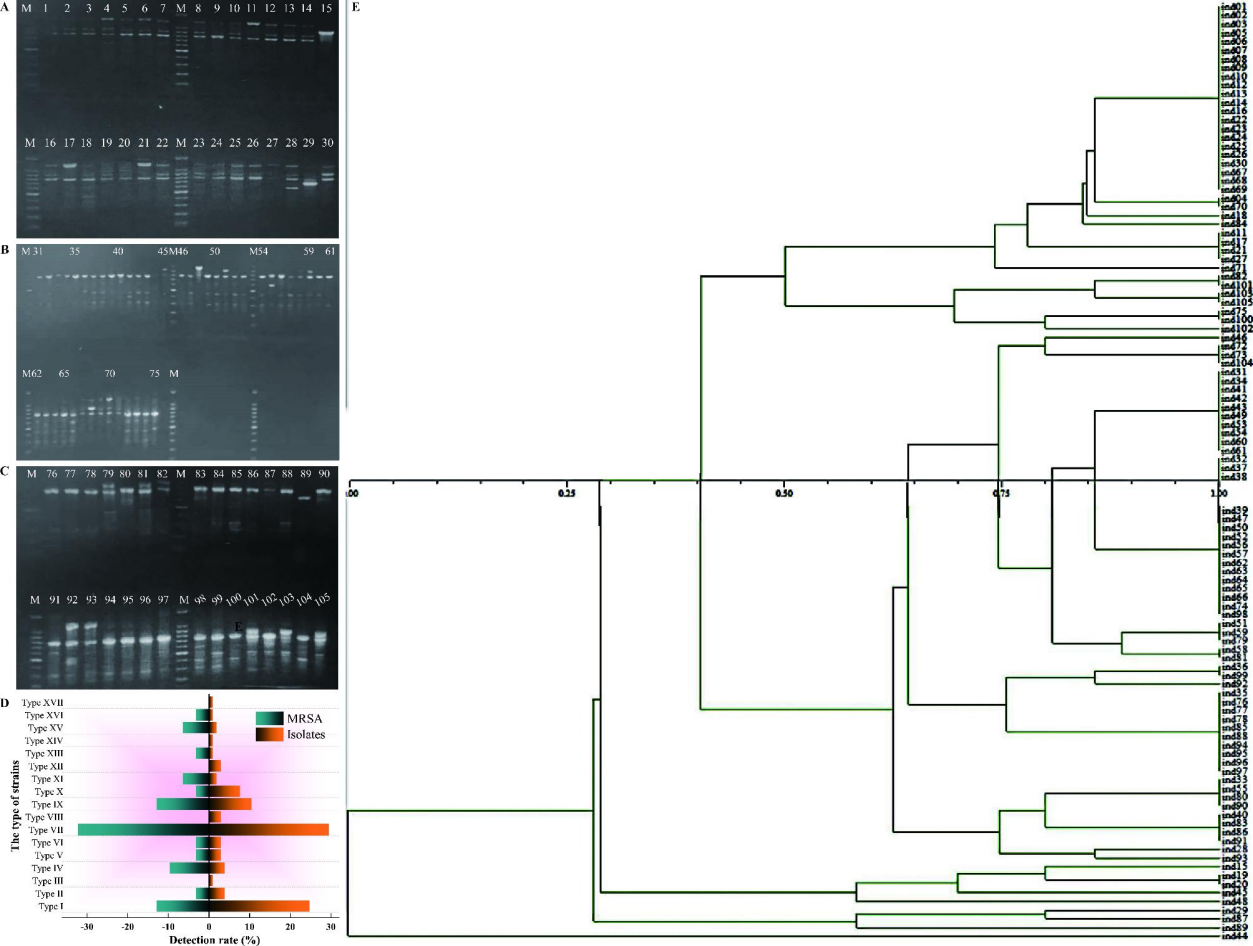
Results of RAPD electrophoresis from the isolates 1–30 (**A**); 31–75 (**B**); 76–105 (**C**). The 17 kinds of gene types among 105 *S. aureus* strains (**D**) analyzed by NTSYS cluster analysis (**E**)

The gene homology of 105 strains of *S. aureus* were analyzed by RAPD technique (Fig. 6). The DNA fingerprints showed 2–6 distinct bands and the size of the product was between 200 and 1500 bp, as shown in Fig. 6A **~ C**. According to the position and number of bands in the RAPD electrophoretic map, similarity at 80% was set as the boundary. By NTSYS cluster analysis (Fig. 6E), 105 strains of *S. aureus* were divided into 17 types (Fig. 6D). The data indicated that the majority of *S. aureus* strains were type VII (29.52%), type I (24.76%) and type IX (10.48%), whilst the majority of MRSA were type VII (32.26%), type I (12.90%) and type IX (12.90%) (Table 6).

**Table 6:** RAPD homology analysis of stain types

Type	Isolates (n = 105)	MRSA (n = 31)
Type I	24.67%	12.90%
Type II	3.81%	3.23%
Type III	0.95%	0%
Type IV	3.81%	9.68%
Type V	2.86%	3.23%
Type VI	2.86%	3.23%
Type VII	29.52%	32.26%
Type VIII	2.86%	0%
Type IX	10.48%	12.90%
Type X	7.62%	3.23%
Type XI	1.90%	6.45%
Type XII	2.86%	0%
Type XIII	0.95%	3.23%
Type XIV	0.95%	0%
Type XV	1.90%	6.45%
Type XVI	0.95%	3.23%
Type XVII	0.95%	0%

## Discussion

Studies have shown that *S. aureus* is highly contagious even with few amounts in patient wound, only 15 cells of *S. aureus* introduced into experimental lesions were enough to be infected [44]. The relatively high virulence and pathogenicity endowed *S. aureus* with a special place in the environment [45]. The people with low immunity are easily infected, especially those patients with damaged skin barrier, and these patients were mainly distributed in Burn and Trauma Departments [46, 47]. In this study, 33 strains of *S. aureus* were detected in the patients occupied in the Burn Department with the highest detection rate, and 26 strains were detected in Trauma Department with the second detection rate. Besides, the majority infections in this work were community-associated (64.76%), which may ascribe to the larger arsenals of virulence factor-encoding genes in CA strains and enabled the transmission among individuals [48]. It was reported that the CA-MRSA often caused severe invasion infections and a wide spectrum of clinical diseases, accounted for a large proportion of the increased disease burden [49].

This survey showed that MRSA caused 29.52% infected wound in patients, which was lower than the data of China Bacterial Resistance Surveillance (CHINET) and the most of other studies [44, 50–52]. The resistance rate of MRSA to the Erythromycin, Clindamycin, Ciprofloxacin, Levofloxacin and Moxifloxacin was significantly higher than that of MSSA strains. The resistance of isolated strains to Nitrofurantoin, Tegacycline, Vancomycin, Teicoplanin, Linezolid and Quinu/Dafoptin could not be found. These results were generally consistent with CHINET, in which the resistance rate of MRSA to most antimicrobial agents was significantly higher than that of MSSA strains, but the resistance rate of MRSA to Trimethoprim-sulfamethoxazole was lower than that of MSSA except for in urine specimens (20.5% and 15.5%) [52]. The resistant strains to Vancomycin could not be found neither MRSA nor MSSA strains in this survey. However, with the extensive use of Vancomycin in clinics, Vancomycin-insensitive and resistant *S. aureus* have been reported one after another [53].

The difference in drug resistance rate of *S. aureus* between CA and HA group was not conspicuous, and similar results was found in the study of Dai Harada but dissimilar with the work of Liesbet [50, 54, 55]. Dai Harada concluded that there was few difference between the groups and found that the antimicrobial susceptibilities was coincidence with the antibiogram change of the HA-MRSA population, affected by the change in the genotype of HA-MRSA [55]. Lisebet found there was obvious difference of drug resistance between CA and HA group [54]. The varieties among these surveys may due to the distributions of geographic region. In this work, the consciousness of infection and control was rooted in the staff, and billions of patients were transferred from lower-level hospitals who had been infected with *S. aureus*, so the distinct difference in drug resistance between CA and HA groups could not be found.

SOFA score is generally used for prognosis of an undesirable disease, especially sever systemic infections of bacteremia and sepsis. Previous literatures shown that patients with MRSA infection had higher SOFA score [56, 57]. The results were consistent with our results. The median of SOFA score in MRSA was higher than that of MSSA, which scored 2 and 1 respectively. The phenomenon could be ascribed to the fact that the MRSA carries PVL gene, the smaller SCC*mec*, and continuously secretes endotoxins and exotoxin, disturbing the immune defense of host and disrupting the systemic barrier. The differences in the SOFA scores between the CA and HA group were significant, result from the population composition between the groups. In HA group, patients were older and with more underlying diseases. However, in CA group, the patients were dominated by young people who recovered relatively quickly. Consequently, the median SOFA score in CA group were lower than that of HA group.

The hospitalization length of MRSA inpatients was longer than that of MSSA, the median of which were 22 days and 15 days, respectively. In a randomized controlled trial, in the US patients with MRSA who were alive at hospital discharge, the median length of stay was 10 days, while the MSSA was 7 days [58]. Compared with the US, the duration in hospital was longer in this survey, due to the gap of medical resource among countries and regions. Besides, the high degree of antibiotic resistance and high mortality of MRSA, which complicated the therapeutic regime and increased the financial burden on medical resources and patients. The median length of stay in CA group (14 to 30 days), was longer than that of HA infections (7 to 14 days). An opposite trend was found in a multi-center nested case-control study, which showed that HAI had longer stay in hospital, with an average of 5 days [59]. However, the workers in this study did not distinguish the pathogens from all infections. The hospital locations and public health conditions were also the reasons for difference between the report and our results.

By NTSYS cluster analysis, 105 strains of *S. aureus* were divided into 17 distinct RAPD types, indicating the diversity of *S. aureus* genotype in this hospital. It was presumed that the most isolates were from the patients themselves with high risk of cross transmission between each other. Various solid surfaces could also be contaminated by MRSA infections with diverse genotypes, allowed the dissemination of bacteria within the hospital environment [60]. Additionally, the strains can be transferred from person to person or from person to frequently touched objects in the nosocomial environment [61]. Consequently, hospitalization duration should be shortened if possible, and specialized isolation precautions should be strictly exerted to prevent cross-infection.

These findings revealed the prevalence and clinical characteristic of *S. aureus* strains collected in the First Affiliated Hospital of Wenzhou Medical University in 2018. Due to the limited sample size, it would be difficult to be used as fully representative data for antibiotic resistance analysis and genetic analysis of pathogens identified in one hospital.

## Conclusion

In summary, this study revealed the clinical epidemiological characteristics of *S. aureus* stains in the First Affiliated Hospital of Wenzhou Medical University in 2018, including the prevalence and the antimicrobial susceptibility patterns of *S. aureus* stains. The results from the investigation highlights the need for continuously monitoring the trend of *S. aureus* and providing the effective management for prevention and infections of *S. aureus* in China.

## Data Availability

All data generated or analyzed during this study are included in this published article. The raw data used in the current study will be available from the corresponding author on reasonable request from Xiaoya Jin (jinxy0001@163.com).

## List of abbreviations

*S. aureus*: *Staphylococcus aureus*
RAPD: the random amplified polymorphic DNA typing
HAI: hospital-acquired infections
CAI: community-acquired infections
PBP2a: penicillin-binding protein 2a
CDC: Center for Disease Control and Prevention
PVL: the Panton-Valentine leucocidin
CLSI: Clinical and Laboratory Standards Institute
MALDI-TOF-MS: the matrix-assisted laser desorption ionization-time off-light mass spectrometry
UPGMA: unweighted pair group method with arithmetic mean
*S*_*D*_: Dice coefficient
SOFA: the Sequential Organ Failure Assessment
*SCC*: staphylococcal cassette chromosome
MST: Multilocus Sequence Typing
